# Contextual adaptation of the Personnel Evaluation Standards for assessing faculty evaluation systems in developing countries: the case of Iran

**DOI:** 10.1186/1472-6920-9-18

**Published:** 2009-04-28

**Authors:** Soleiman Ahmady, Tahereh Changiz, Mats Brommels, F Andrew Gaffney, Johan Thor, Italo Masiello

**Affiliations:** 1 Department of Learning, Informatics, Management, and Ethics, Karolinska Institutet, Stockholm, Sweden; 2 Educational Development Center, Urmia University of Medical Sciences, Urmia, Iran; 3 National Public health Management Center, Tabriz University of Medical Sciences, Tabriz, Iran; 4 Medical Education Research Center, Isfahan University of Medical Sciences, Iran; 5 Medical Management Centre, Department of Learning, Informatics, Management, and Ethics, Karolinska Institutet, Stockholm, Sweden, and Department of Public Health, University of Helsinki, Finland; 6 Vanderbilt University School of Medicine, Nashville, Tennessee, USA; 7 Medical Management Centre, Department of Learning, Informatics, Management, and Ethics, Karolinska Institutet, Stockholm, Sweden; 8 Centre for Medical Education, Department of Learning, Informatics, Management, and Ethics, Karolinska Institutet, Stockholm, Sweden

## Abstract

**Background:**

Faculty evaluations can identify needs to be addressed in effective development programs. Generic evaluation models exist, but these require adaptation to a particular context of interest. We report on one approach to such adaptation in the context of medical education in Iran, which is integrated into the delivery and management of healthcare services nationwide.

**Methods:**

Using a triangulation design, interviews with senior faculty leaders were conducted to identify relevant areas for faculty evaluation. We then adapted the published checklist of the Personnel Evaluation Standards to fit the Iranian medical universities' context by considering faculty members' diverse roles. Then the adapted instrument was administered to faculty at twelve medical schools in Iran.

**Results:**

The interviews revealed poor linkages between existing forms of development and evaluation, imbalance between the faculty work components and evaluated areas, inappropriate feedback and use of information in decision making. The principles of Personnel Evaluation Standards addressed almost all of these concerns and were used to assess the existing faculty evaluation system and also adapted to evaluate the core faculty roles. The survey response rate was 74%. Responses showed that the four principles in all faculty members' roles were met *occasionally *to *frequently*. Evaluation of teaching and research had the highest mean scores, while clinical and healthcare services, institutional administration, and self-development had the lowest mean scores. There were statistically significant differences between small medium and large medical schools (p < 0.000).

**Conclusion:**

The adapted Personnel Evaluation Standards appears to be valid and applicable for monitoring and continuous improvement of a faculty evaluation system in the context of medical universities in Iran. The approach developed here provides a more balanced assessment of multiple faculty roles, including educational, clinical and healthcare services. In order to address identified deficiencies, the evaluation system should recognize, document, and uniformly reward those activities that are vital to the academic mission. Inclusion of personal developmental concerns in the evaluation discussion is essential for evaluation systems.

## Background

Performance evaluation of university faculty has received increased attention in recent years [1-4]. The intention is to improve faculty performance, professional development and healthcare practice. Given the importance of performing faculty evaluation in a reliable and valid manner, many medical schools today are searching for ways to effectively and constructively evaluate faculty performance and ways to implement evaluation systems that are fair and standardized across departments [4-6].

In this study "faculty evaluation" is defined as formal measures made by academic authorities in medical schools to assess the academic performance of faculty members. These assessments result in a judgment about faculty performance and may be followed by either positive or negative promotion decisions. Thus, *faculty evaluation system *refers to the inter-related elements and processes that produce evaluation data, and provide useful performance feedback. By this definition, a comprehensive faculty evaluation system proposes to systematically and fairly document and evaluate academic activities, ie, all activities related to teaching, research, administration and services [7-10].

Continuous professional development of faculty has also become an issue among authorities, policymakers, and leaders of academic institutions and healthcare delivery organizations. The reason for this is that faculty evaluation could provide relevant and reliable information on which to base promotion, tenure, merit pay and/or for personal growth and improvement[6,10]. However, for optimal effectiveness, faculty evaluation systems should be linked to faculty development programs.

A variety of approaches on faculty evaluation have been reviewed by Bland et al[4]. For instance, the authors present a goal-based approach in which faculty members would prepare annual goals and be evaluated at the end of the year on goal accomplishment. Colbeck[11] presents an integrated faculty work approach (integration of two methods for evaluating: workload report and annual reports method). Another approach, is to apply an institution-wide comprehensive system for faculty evaluation[7,12]. Arreola states[12] that there should be four key elements for the design of a faculty evaluation system: (i) establishing a good fit between the system and the environment, (ii) securing strong faculty involvement, (iii) providing not only feedback on faculty members' performances but also (iv) developing opportunities to improve their future performance. So, a comprehensive faculty evaluation approach should take a multi-dimensional view in which information is provided by students, colleagues, academic administrators, and faculty members themselves as self-evaluators[2,13-15].

Developing and implementing effective faculty evaluation systems is challenging[7]. Many stumbling blocks are discussed in the literature, including: a dominance of administrative summative purposes in faculty evaluations, faculty resistance, over-reliance on student opinions, over-reliance on self-report data, and administrative disinterest[6]. Of those, two are believed by Arreola[5,16] to be major ones: faculty resistance and administrators' apathy. Also several solutions have been proposed. For instance, developing and using a comprehensive faculty evaluation system by considering all academic activities of faculty members[7], or also adopting the Personnel Evaluation Standards, which can address the technical process (building reliable and valid measurement tools) and the political process (building consensus around shared values)[17].

Personnel Evaluation Standards (hereafter referred to as the Standards) provide a systematically developed and widely endorsed basis for evaluation of personnel evaluation systems [17-20]. Recognized Standards are those issued by the Joint Committee on Standards for Educational Evaluation. These Standards are organized into four basic principles of sound evaluation: Utility (how to make evaluations more useful and more often used), Feasibility (how feasibly to conduct evaluations in the real world where little can be controlled and politically difficult situations abound), Propriety (how to ensure propriety in all aspects of the evaluation), and Accuracy (how to promote accurate and dependable evaluation). These are described as "four main concerns about any evaluation", and each incorporates several standard measures[19,20].

The Standards have been used for evaluating the qualifications and performance of teachers and other educators[17,21,22]. Hence, universities and other educational institutions may use the Standards to develop a checklist of basic requirements of their evaluation systems, both to assure that they are sound and to guide needed or desirable improvements. Thus, adaptation of the Standards for faculty evaluation systems in medical schools can assure a systematic approach, and help acknowledge faculty's multiple academic roles.

While there is a growing concern about performing effective and efficient faculty evaluations in medical schools of developing countries, little is reported on how best to do it. In the case of Iran, we are unaware of any recent publications discussing systematic faculty evaluation. In Iran, the national integration of medical education into healthcare services, under the Ministry of Health and Medical Education, has added more challenges to faculty, including heavy responsibilities for healthcare delivery[23,24]. Consequently, faculty roles have become more complex[25]. How to address these multiple roles in faculty evaluation and development remains an open question. Furthermore, there is limited empirical data on attempts to link faculty development activities to faculty evaluation in order to continuously maintain and improve faculty performance and achieve high quality healthcare.

The current faculty evaluation system in Iranian medical schools is based on a highly refined checklist with questionnaires related to the quantity and quality of teaching in classroom (classroom instruction/effectiveness). It relies also heavily on students opinions. On the other hand, decisions on faculty promotion and tenure mainly depend on the quantity and quality of their scientific publications. So, it seems that other important components and responsibilities of faculty members are relatively neglected or weighted differently[1,4,8], as for instance the performance of clinical and community healthcare delivery[24].

A recent study on the faculty development system in Iranian medical schools showed that most schools lack an integrated system that provides medical faculty with relevant and appropriate opportunities for professional development. One of the major challenges was the poor linkage between faculty development and evaluation systems[26]. The main reason behind this problem might be a failure of the evaluation system. Without a standardized, systematic and fair approach to faculty evaluation, the process becomes a threat and the antithesis of effective evaluation and development[5]. The necessity and challenges related to establishing such a linkage have been shown before[27]. This not only requires accountability from both medical schools leaders and faculty members, but also necessitates a well established system for faculty evaluation and development.

The purpose of this study is to develop a foundation for the design of a comprehensive national approach to medical faculty evaluation by examining i) the views on evaluation of faculty members in managerial and leadership position in Iranian medical universities, ii) which areas and components of faculty work should be evaluated, and iii) whether adaptation of the Standards will enable evaluation that addresses all roles and responsibilities of faculty members.

## Methods

### Research Design

Our research strategy utilized a three-step evaluation. First, senior faculty input was sought in order to learn about faculty evaluation in Iran. Second, the data were used to map against the Standards and adapt a new instrument to the Iranian context. And third, a broader perspective then was explored through a national survey. Hence, this study employed methodological triangulation with qualitative and quantitative data collection.

### Semi-structured interviews

Semi-structured interviews were used to explore knowledge and perceptions of senior faculty leaders and to gain a better understanding about the current faculty evaluation process in Iran. An interview protocol was developed using a number of questions designed to allow respondents to focus on the topics and issues that they considered most important about their experiences in faculty evaluation. In this qualitative approach, randomization to select informants was not a primary concern, so a strategic or purposive sample of key informants was used to interview senior faculty leaders who have extensive knowledge, and experience performing faculty evaluations. In addition, maximal variation sampling was used by selecting interviewees with different responsibilities and levels of expertise in different levels of medical universities and the Ministry of Health and Medical Education. All participants were approached personally by SA, consented to be interviewed and to the audio-taping of the interview. All tape-recorded interviews were transcribed verbatim by the same author. Analysis was performed using qualitative content method. The transcripts were analyzed and checked for accuracy by sending them back to the interviewees who were asked to report whether the text gave an accurate representation of the interview ('member checking'). In addition, to validate the findings transcripts were read and coded independently by two researchers (SA and a third-party investigator). Any difference was resolved by subsequent discussion. To reduce the data we constructed *meaning units *throughout the analysis process. Then we extracted a short description of meaning units. These descriptions were then further condensed, so that categories were developed during the iterative process of analysis and also discussion among the researchers in order to reach consensus.

Data saturation was reached when no new information was obtained and after interviewing 21 senior faculty leaders. The interviews served two major purposes. First, they provided the background and insights used in constructing an instrument mapped against the Standards. Second, the interviews were data sources that we analyzed to suggest themes and important aspects of faculty members' experiences which might not be addressed in the Standards.

### Consensus decision-making group

The issues raised by the interviewees were many and complex to be handled by one person. Therefore, agreement was reached by conducting consensus decision-making between experts in the field of medical education. The purpose of the decision-making group was to present and discuss the different views raised by the interviewees and finally reach consensus on which statements of the Standards fit the interview data.

### Adaptation of the Standards and development of the instrument

Adaptation of the Standards was conducted because of their widespread use in evaluation of personnel evaluation systems[21,22,28]. Some of the standard measures were dropped as not all were equally applicable to our context (see Additional file 1). *Propriety *includes seven standard measures, but we used only six; for *accuracy*, we used seven standards out of eleven; for *feasibility*, two out of three; and for *utility *all six standard measures were used. Finally, the instrument developed from interviews and the consensus decision-making group covered 21 standard measures distributed in 27 statements. Each statement was expected to provide information in five separate areas of faculty efforts, including teaching, research and scholarly activities, clinical and healthcare services, institutional administration, and self-development. A 5-point scale provided the answers: "Don't know", "Never", "Occasionally", "Frequently", and "Always" with the scores of 0, 1, 2, 3, and 4 respectively (see Additional file 2).

To get broad data and solicit the opinion of respondents, the instrument included a short survey of three open-ended questions asking participants to state the extent to which they thought that the current evaluation system facilitated faculty members' improvement. They were asked to write how faculty evaluation system could better support faculty and leaders in achieving the institution's mission. They also were asked to mention some barriers and limitations of the current approach to faculty evaluation and to give suggestions for how to conduct a fair and effective evaluation system.

The instrument included demographic variables, as well addressing academic rank, departmental affiliation, current managerial or leadership position, current roles, and experience. The instrument underwent all the necessary steps of development in order to test its validity and reliability in the new context. To address the validity of the questionnaire we had a formal group discussion with six experts from the medical university of Isfahan with experience in the field of faculty evaluation. This group discussion provided credibility to the process of questionnaire development. A pilot study with 20 faculty members from two medical schools was conducted as well. Additionally, we examined whether the statements would have been better written in the form of statements or questions, and the appropriate response options with their respective scale. After pilot testing the statements were turned into questions and were revised to improve clarity and face validity. Cronbach's alpha was calculated to estimate the reliability of the instrument and internal consistency of all questions. The Cronbach's coefficient α was 0.98.

### Sampling and data collection

To represent all 40 public Iranian medical schools, we surveyed a national sample of 345 faculty members in different leadership and administrative positions, in twelve medical schools of different sizes. The sample selection for each school's faculty was stratified into large, medium and small size schools (sizing is based on the number of faculty members, number of students, conducting or not conducting postgraduate educational programs, carrying out residency or subspecialty programs etc), with four schools in each stratum. Respondents were head of departments and faculty members with administrative and managerial positions, as they are expected to have sufficient contact with faculty evaluation systems. Survey packages were mailed to participants. The questionnaire was coded to location so that the requested numbers of surveys were obtained for each site. The survey itself was completely anonymous. Subjects were advised in a cover letter that participation was optional and that consent was implied by the completion and return of the survey. They were instructed to answer specifically regarding their own institution's faculty evaluation processes at that time. Responders were asked also to return the completed survey to their EDC. EDC's personnel carried out a series of follow-up activities, such as telephone reminders and repeated mailing of the full package, to encourage participation and increase the response rate. In addition, a liaison was recruited in each school to follow up and encourage survey completion.

### Data entry and analysis

Data from the questionnaire were manually entered into a spreadsheet and afterward checked for accuracy. Data were analyzed using SPSS 11.5 (Statistical Package for Social Sciences). To identify differences, data were analyzed using one-way ANOVA. Duncan's post hoc analysis was applied when appropriate. One-way ANOVA was also used to assess if differences existed by rank, roles of faculty and size of medical schools.

### Ethical considerations

The study was designed and developed as a joint project between the department of Learning, Informatics, Management and Ethics at the Karolinska Institute, Sweden and Medical Education Research Center at medical university of Isfahan, and the National Public Health Management Center (NPMC). Data collection has been performed on a nation-wide level in all public Iranian medical schools. So ethical approval was sought and obtained from the national ethics committee of the Ministry of Health and Medical Education in Iran.

## Results

### Qualitative method findings

Interview data were coded, categorized, and tabulated using qualitative content analysis. The analysis yielded a number of statements which then identified four categories (Table 1), including:

**Table 1:** Categories (A) developed from the analysis of the interviews; description statements (B) resulted from the interviews and making up the categories; and frequencies of the respondents' comments to the open-ended questions (C) which matched the statements from the interviews

**A**	**B**	**C**	
**Category**	**Description**	**Respondents Comment**	
		
		**Percent**	**Number**

Purpose and objectives of evaluation	It has relatively minimal effect on mission achievement	75	191 out of the 254
	
	Evaluation exists in isolation from development	78	198 out of the 254
	
	Evaluation did not provide enough opportunity for promotion, retention, and tenure decisions	50	127 out of the 254
	
	Faculty members do not recognize the benefit of evaluation	60	153 out of the 254
	
	Faculty evaluation process has not been perfectly designed to assist the institution in attracting faculty members, helping them reach their potential, and rewarding their proficiency	71	180 out of the 254

Criteria and standards of evaluation	Objectives agreed to are changed, so that they do not become the bases for the criteria to be applied in subsequent reviews	58	147 out of the 254
	
	Lack of criteria and standards for evaluation	79	201 out of the 254
	
	There was no differentiation between competent and incompetent faculty members	46	117 out of the 254
	
	The designed guideline are not always complying with standards	65	165 out of the 254

Area of faculty evaluation	There is no multiple role approach in evaluation, so that faculty were not evaluated for all components that influence their performance	79	201 out of the 254
	
	Little weight is given to clinical and community healthcare service	42	107 out of the 254
	
	There is wide disagreement within institutions and departments concerning the importance given to teaching, research, clinical and administrative services	63	160 out of the 254
	
	In spite of potential advantages of program integration, there was no demand for applying these opportunities	39	99 out of the 254
	
	Scholarship goals neither specific nor fairly measurable	64	163 out of the 254
	
	Over reliance on student evaluation of classroom teaching evoked negative responses on faculty (Student-centered evaluation)	81	206 out of the 254

Administration and procedures of faculty evaluation	Due to faculty resistance evaluation somehow fails. Faculty resists evaluation because they do not trust the reasoning behind it	49	124 out of the 254
	
	The tools for gathering faculty work data are not standardized	67	170 out of the 254
	
	There are possibilities for subjective evaluation	59	150 out of the 254
	
	Due to some insufficiency in evaluation system, feedback to faculty members is not provided	69	175 out of the 254
	
	Evaluation process is somehow unclear and non-directive	61	155 out of the 254
	
	Departments are not involved	44	112 out of the 254
	
	Faculty are frustrated because evaluations take time but yield little benefit	56	142 out of the 254
	
	The system does not provide adequate incentives (merit) for excellent performers	63	160 out of the 254
	
	They have not been treated fairly in the process	51	130 out of the 254

1. Purpose and objectives of evaluation;

2. Criteria and standards of evaluation;

3. Area of faculty evaluation; and

4. Administration and implementation of evaluation.

The categories and the statements making them up were subsequently analyzed against the open-ended questions of the survey responses. The percentages of the comparable comments are shown in Table 1, column C. The most frequent comments under the purpose and objectives of evaluation category were: 198 respondents (78%) wrote that evaluation is done in isolation from development, has minimal effect on mission achievement (191; 75%), and is not designed to attract, support or reward faculty members (180; 71%). Under the criteria and standards of evaluation category, 201 (79%) concurred that there is a lack of criteria and standards for evaluation. Under the area of faculty evaluation, 206 (81%) noted over reliance on student evaluation, while 201 (79%) agreed that there is no multiple role approach in evaluation. Lastly, under the administration and procedures of faculty evaluation category, 175 respondents (69%) concurred that the evaluation systems do not provide necessary feedback to faculty (Table 1).

### Survey findings

The number of returned questionnaires was 273 out of 345, 19 incomplete surveys were discarded. Consequently, 254 cases were considered for analysis (74% response rate). The respondents' positions were: 9 (3.5%) university vice chancellors, 8 (3.1%) school deans, 29 (11.4%) vice deans, 179 (70.5%) heads of departments and 29 (11.4%) educational directors or other senior administrators. Table 2 lists the characteristics of the respondents.

**Table 2:** Characteristics of respondents based on their position, rank, and school size

***Characteristics***	
**Respondent's position**	
Vice-Chancellor	9 (3.5%)
Dean	8 (3.1%)
Vice-Dean	29 (11.4%)
Department Head	179 (70.5%)
EDC Director & Educational Director	29 (11.4%)
**Respondent's school type**	
Large Size	108 (42.5%)
Middle Size	100 (39.4%)
Small Size	46 (18.1%)
**Respondent's experience in current position**	
Less than 5 years	60.7%
5–10 years	21.2%
More than 10 years	18.1 (%)
**Respondent's Academic Rank**	
Professor	19 (7.5%)
Associate professor	60 (23.6%)
Assistant professor	154 (60.6%)

The 21 standard measures were assessed in the same order as they appear in the original document and are shown in Additional file 1. As the full document has been published previously[19,20], only a summary statement of results is provided to highlight the key findings. Additional details are provided in Table 3.

**Table 3:** Frequency of respondents in five scale based on principles of sound evaluation and Standards

		Percent addressed and met				
***Category (Principle)***	***Personnel Evaluation Standards***	Never = 1	Occasionally = 2	Frequently = 3	Always = 4	No Idea = 0
**Propriety**						
	P1. Service Orientation	34.6	24.8	40.6	.00	.00
	P2. Appropriate Policies and Procedures	16.25	31	25.23	15.87	11.65
	P3. Access to Evaluation Information	5.75	20.42	23.38	20.6	29.68
	P4. Interactions with Evaluatees	7.48	19.44	29.5	22.6	20.98
	P5. Balanced Evaluation	25.28	31.5	21.26	9.68	12.28
	P6. Conflict of Interest	13.54	28.28	31.04	11.18	15.98
**Utility**						
	U1. Constructive Orientation	11.38	30.68	28.54	13.62	15.78
	U2. Defined Uses	23.02	28.38	16.46	10.32	21.82
	U3. Evaluator Qualifications	11.51	26.11	27.73	16.96	17.69
	U4. Explicit Criteria	12.6	28.88	26.78	24.88	6.86
	U5. Functional Reporting	13.57	27.02	29.2	12.45	17.76
	U6. Professional Development	16	27.76	33.61	6.63	16
**Feasibility**						
	F2. Political Viability	24.4	28.52	19.28	10.54	17.26
	F3. Fiscal Viability	9.38	28.34	24.16	12.84	25.28
**Accuracy**						
	A1. Validity Orientation	20.64	31.8	15.36	6.62	25.58
	A2. Defined Expectations	12.87	30.12	28.95	17.44	10.62
	A4. Documented Purposes and Procedures	5.72	19.28	26.3	19.28	29.42
	A5. Defensible Information	12.28	24.24	29.02	12.2	22.26
	A7. Systematic data control	6.5	17	32.5	18.9	25.1
	A8. Bias Identification and Management	6.54	16.92	32.22	18.96	25.36
	A10. Justified Conclusions	21.8	26.06	17.96	23.4	10.78

For the first propriety standard of *service orientation*, 40% of respondents stated that it was frequently met, while 35% responded that it was never met. For *appropriate policies and procedures*, about 41% stated that it was frequently or always evaluated, while about 47% stated that it was never or only occasionally assessed. For *balanced evaluation *more than half of the respondents stated it was never or occasionally met. Regarding the Utility category, *constructive orientation*, responses were equally split (40%, 42%) between frequently-always met and occasionally-never met. For *professional development *more than a quarter of respondents (34%) stated that it was frequently met but for 16%, it was never met. Concerning the Accuracy standard, more than half of the faculty members stated that *validity orientation *was never or occasionally met, while another quarter provided no estimate. For *systematic data control*, more than half of faculty members stated that it was frequently or always performed, while a quarter of them did not know.

When considering mean scores of the four basic principles of sound evaluation in five domains of faculty members' roles (Table 4), we found that all mean scores were between "1" and "2", meaning that the corresponding standards were "never" or "occasionally" met. There were no mean scores around the "frequently" or "always" options. Within the different roles of faculty members, teaching and research efforts had the highest mean score (1.82 ± 0.65 and 1.71 ± 0.72 respectively); while clinical and healthcare services, institutional administration, and self-development activities were least likely to have been evaluated.

**Table 4:** Mean scores of the four basic principles of sound evaluation based on academic roles and activities

***Principles***	**Mean (*Std Dev*)**					
	Teaching	Research	Clinical & healthcare Service	Administration	Self-devel.	Total

Propriety	1.82 (± 0.65)	1.71(± 0.72)	1.46(± 0.81)	1.48(± 0.74)	1.22(± 0.80)	1.55(± 0.64)
Utility	1.67(± 0.61)	1.61(± 0.68)	1.44(± 0.67)	1.38(± 0.68)	1.11(± 0.73)	1.48(± 0.63)
Feasibility	1.47(± 0.84)	1.37(± 0.86)	1.32(± 0.87)	1.21(± 0.89)	1.11(± 0.88)	1.30(± 0.79)
Accuracy	1.73 (± 0.65)	1.58 (± 0.69)	1.50 (± 0.76)	1.42 (± 0.72)	1.15 (± 0.78)	1.48(± 0.63)

One-way analysis of variance examined the relationship between school size and the four basic principles of sound evaluation (Table 5). For the *Accuracy *principle, for example, there was a statistically significant difference between small, middle or large size schools (p ≤ .000, F = 8.95) so that Duncan's Post Hoc analysis revealed statistical significance at the .05 level and identified differences between small size schools from other (middle and big size schools). Meanwhile, Duncan post hoc analyses for multiple comparisons showed that there were statistically significant differences for all the basic principles observed in small size schools than middle and big size schools.

**Table 5:** Summary table of ANOVA for comparison between school size and principles of sound evaluation

	Large Size		Middle Size		Small Size		Total		
				
	Mean	Std dev.	Mean	Std dev.	Mean	Std dev.	Mean	F	P*
Propriety	1.51	± .58	1.42	± .65	1.7	± .68	1.55	5.51	.005
Utility	1.41	.52	1.29	.63	1.68	.62	1.44	7.88	.000
Feasibility	1.37	.75	1.00	.75	1.55	.82	1.30	9.26	.000
Accuracy	1.48	.59	1.36	.63	1.76	.64	1.48	8.95	.000

*P < 0.05 Significant

One-way ANOVA also showed that, irrespective of medical school size and educational programs, there were statistically significant differences in mean scores between the respondents' job position and the principles of evaluation (Table 6). There were statistically significant differences between mean scores assigned by department heads and school deans, vice chancellors and other directors, in almost all principles of evaluation. For example in the *Utility *category, the mean score for department heads was 1.38 but for vice chancellors and directors it was 1.5 and 1.86, respectively (p ≤ .003, F = 4.2) so that Duncan's Post Hoc analysis revealed statistical significance at the .05 level and identified differences between *department heads *from other job positions.

**Table 6:** Summary table of ANOVA for comparison between faculty member perceptions and principles of sound evaluation

	Dept. Heads		Deans		Directors		Vice Chancellor		Total		
					
	Mean	Std dev.	Mean	Std dev.	Mean	Std dev.	Mean	Std dev.	Mean	F	P*
Propriety	1.49	± .64	1.50	± .66	1.97	± .71	1.53	± .71	1.55	3.60	.007
Utility	1.38	.58	1.4	.53	1.86	.68	1.50	.67	1.44	4.20	.003
Feasibility	1.2	.79	1.19	.69	1.8	.79	1.5	.82	1.30	5.11	.001
Accuracy	1.41	.61	1.57	.54	1.88	.74	1.57	.70	1.48	3.60	.007

*P < 0.05 Significant

## Discussion

The initial interview findings showed how faculty members relate to and think about the evaluation systems used at their institution. The categories emerged from the semi-structured interviews highlights the difficulties and limitations of existing faculty evaluation systems within four large areas, purpose and objectives, criteria and standards, area, and administration of evaluation.

The categories were compared with the open-ended questions of the survey which confirmed faculty's concerns regarding faculty evaluation. The results demonstrate a strong belief that medical school evaluations should address faculty members' needs, help performance improvement, yield defensible personnel decisions, and effectively provide high quality healthcare services and medical education. Furthermore, the analyses revealed that current faculty evaluation systems suffer from major barriers and limitations. They have shortcomings with regard to defining, designing, collecting, analyzing and reporting. Although the investigated medical schools have acknowledged efforts in the establishment of faculty evaluation systems, there is still a need for a sound faculty evaluation.

However, if faculty evaluation is to be convincing and fair, it should be underpinned by standard measures. To fully explore these issues, we adapted the Standards to the Iranian medical schools context. According to the new adaptation we were able to look at faculty evaluation system considering the broad range of faculty members' roles. The mean scores of the four basic principles of sound evaluation (utility, propriety, feasibility and accuracy) show that evaluation systems do not correspond to major faculty responsibilities and are inconsistently addressed during faculty evaluations. The evaluation process mostly relies on research and teaching activities. Neglecting the other aspects of faculty members' roles yields an incomplete and unbeneficial evaluation[1,11,27,29]. Systematic evaluation of all faculty academic activities and roles is vital to creating evaluation accountability, especially and more broadly to the fulfillment of academic institutions' mission[15,27]. The results of this study may elucidate potential features to be changed in the faculty evaluation system in Iran.

We showed that application of the adapted Standards confirms and complement the results of the qualitative data. For that matter, the adapted Standards could help detect and correct deficiencies. At the same time, they offered educators, administrators, and other policy makers widely shared principles for reviewing existing approaches, for developing and assessing new or improved approaches, for guiding these approaches to work beneficially, and for defending sound approaches against legal and other challenges. We also are aware that involving all stakeholders such as administrators, students and other staff in the same approaches could benefit further the evaluation systems.

To compare some findings of this study with the broad international picture, we noted similarities among the faculty evaluation systems including: i) Academic institutions accept standard-based evaluations and also adaptation of the appropriate Standards as the foundation for reforming their systems for assessing the organization's evaluation system[17]. ii) The perception that current academic systems add too much pressure to faculty members' workloads, while faculty members are not evaluated against their performing roles and responsibilities. iii) Academic administrators struggled to conduct an effective evaluation system in order to provide enough feedback and opportunity for continuous professional development of their staff [30-33]. Our findings reveal that there is, to some extent, a global problem in several aspects of ongoing faculty evaluation systems that requires academic organizations to conduct sound evaluation systems. In establishing such a faculty evaluation system, the main step is the appropriate adaptation of the Standards.

## Conclusion

Current evaluation systems for medical school faculty do not distinguish between faculty performance and institution performance, even though they should not be isolated from faculty development opportunities. Considering the results of this study we may conclude that faculty evaluation systems in Iranian medical schools congruent with the Standards. In order to address identified deficiencies, the evaluation system should recognize, document, and uniformly and equitably reward those activities that are vital to the academic mission. If evaluation is to be optimizing faculty members' potential contributions, it should be able to assess all aspects of the academic system.

Faculty evaluation system can provide an overview of faculty members' performance that is essential for their professional career. But no all systems are perfect. Academic institutions should be continuously exploring ways to improve their faculty evaluation systems. Finally, this paper is a brief report on a meta-evaluation of faculty evaluation system that provides data-driven suggestions for improving faculty evaluation. Limitations in the current faculty evaluation systems must be kept in mind as future improvements and changes are made. Our analysis not only guides the design of a new approach to faculty evaluation for Iranian medical schools, but also describes an experience of meta-evaluation that could be useful to investigators elsewhere.

## Abbreviations

PES: Personnel Evaluation Standards; EDC: Educational Development Center; ANOVA: Analysis of Variance; KI: Karolinska Institutet; LIME: Learning, Informatics, Management, and Ethics; CME: Centre for Medical Education.

## Competing interests

The authors declare that they have no competing interests.

## Authors' contributions

SA and TC, MB, IM participated in the design of the study and in the interpretation of the data. SA, TC performed the statistical analyses and prepared the manuscript. AG, JT, and IM edited the manuscript. All authors read and approved the final manuscript.

## Pre-publication history

The pre-publication history for this paper can be accessed here:



## Supplementary Material

Additional file 1 **Summary of the Personnel Evaluation Standards checklist.** Click here for file

Additional file 2 A schematic view of the survey instrument. Click here for file
