# Supplementary material for: Contextual adaptation of the Personnel Evaluation Standards for assessing faculty evaluation systems in developing countries: the case of Iran
Source: BMC Med Educ. 2009 Apr 28;9:18. doi: 10.1186/1472-6920-9-18 (PMC2680845; doi:10.1186/1472-6920-9-18)
Supplement: Additional file 1 — Summary of the Personnel Evaluation Standards checklist. [file 1472-6920-9-18-S1.doc]

### Additional file 1: Summary of the Personnel Evaluation Standards

| ***Principle*** | ***Standards*** | Description |
| --- | --- | --- |
| ***Propriety*** | ***P1. Service Orientation*** | **Personnel evaluations should promote sound education, fulfillment of institutional missions, and effective performance of job responsibilities, so that the educational needs of students, community, and society are met.** |
|  | ***P2. Appropriate Policies and Procedures*** | **Guidelines for personnel evaluations should be recorded and provided to the evaluatee in policy statements, negotiated agreements, and/or personnel evaluation manuals, so that evaluations are consistent, equitable, and fair.** |
|  | ***P3. Access to Evaluation Information*** | **Access to evaluation information should be limited the persons with established legitimate permission to review and use the information, so that confidentiality is maintained and privacy protected.** |
|  | ***P4. Interactions with Evaluatees*** | **The evaluator should respect human dignity and act in a professional, considerate, and courteous manner, so that the evaluatee's self-esteem, motivation, professional reputations, performance, and attitude toward personnel evaluation are enhanced or, at least, not needlessly damaged.** |
|  | ***P5. Balanced Evaluation*** | **Personnel evaluations should provide information that identifies both strengths and weaknesses, so that strengths can be built upon and weaknesses addressed.** |
|  | ***P6 Conflict of Interest*** | **Existing and potential conflicts of interest should be identified and dealt with openly and honestly, so that they do not compromise the evaluation process and results.** |
|  | ***P7 Legal Viability*** | **Personnel evaluations should meet the requirements of all federal, state, and local laws, as well as case law, contracts, collective bargaining agreements, affirmative action policies, and local board policies and regulations or institutional statutes or bylaws, so that evaluators can successfully conduct fair, efficient, and responsible personnel evaluations.** |
| ***Utility*** | ***U1. Constructive Orientation*** | **Personnel evaluations should be constructive, so that they not only help institutions develop human resources but encourage and assist those evaluated to provide excellent services in accordance with the institution's mission statements and goals.** |
|  | ***U2. Defined Uses*** | **Both the users and intended uses of a personnel evaluation should be identified at the beginning of the evaluation so that the evaluation can address appropriate questions and issues.** |
|  | ***U3. Evaluator Qualifications*** | **The evaluation system should be developed, implemented, and managed by persons with the necessary qualifications, skills, training, and authority, so that evaluation reports are properly conducted, respected and used.** |
|  | ***U4. Explicit Criteria*** | **Evaluators should identify and justify the criteria used to interpret and judge evaluatee performance, so that the basis for interpretation and judgment provide a clear and defensible rationale for results.** |
|  | ***U5. Functional Reporting*** | **Reports should be clear, timely, accurate, and germane, so that they are of practical value to the evaluatee and other appropriate audiences.** |
|  | ***U6. Professional Development*** | **Personnel evaluations should inform users and evaluatees of areas in need of professional development, so that all educational personnel can better address the institution's missions and goals, fulfill their roles and responsibilities, and meet the needs of students.** |
| ***Feasibility*** | ***F1 Practical Procedures*** | **Personnel evaluation procedures should be practical, so that they produce the needed information in efficient, non-disruptive ways.** |
|  | ***F2 Political Viability*** | **Personnel evaluations should be planned and conducted with the anticipation of questions from evaluatees and others with a legitimate right to know, so that their questions can be addressed and their cooperation obtained.** |
|  | ***F3 Fiscal Viability*** | **Adequate time and resources should be provided for personnel evaluation activities, so that evaluation can be effectively implemented, the results fully communicated, and appropriate follow-up activities identified.** |
| ***Accuracy*** | ***A1. Validity Orientation*** | **The selection, development, and implementation of personnel evaluations should ensure that the interpretations made about the performance of the evaluatee are valid and not open to misinterpretation.** |
|  | ***A2. Defined Expectations*** | **The qualifications, role, and performance expectations of the evaluatee should be clearly defined, so that the evaluator can determine the evaluation data and information needed to ensure validity.** |
|  | ***A3 Analysis of Context*** | **Contextual variables that influence performance should be identified, described, and recorded, so that they can be considered when interpreting an evaluatee's performance.** |
|  | ***A4 Documented Purposes & Procedures*** | **The evaluation purposes and procedures, both planned and actual, should be documented, so that they can be clearly explained and justified.** |
|  | ***A5 Defensible Information*** | **The information collected for personnel evaluations should be defensible, so that the information can be reliably and validly interpreted.** |
|  | ***A6 Reliable Information*** | **Personnel evaluation procedures should be chosen or developed and implemented to assure reliability, so that the information obtained will provide consistent indications of the evaluatee's performance.** |
|  | ***A7 Systematic Data Control*** | **The information collected, processed, and reported about evaluatees should be systematically reviewed, corrected as appropriate, and kept secure, so that accurate judgments about the evaluatee's performance can be made and appropriate levels of confidentiality maintained.** |
|  | ***A8 Bias Identification and Management*** | **Personnel evaluations should be free of bias, so that interpretations of the evaluatee's qualifications or performance are valid.** |
|  | ***A9 Analysis of Information*** | **The information collected for personnel evaluations should be systematically and accurately analyzed, so that the purposes of the evaluation are effectively achieved.** |
|  | ***A10 Justified Conclusions*** | **The evaluative conclusions about the evaluatee's performance should be explicitly justified, so that evaluatees and others with a legitimate right to know can have confidence in them.** |
|  | ***A11 Metaevaluation*** | **Personnel evaluation systems should be examined periodically using these and other appropriate standards, so that mistakes are prevented or detected and promptly corrected, and sound personnel evaluation practices are developed and maintained over time.** |
| ***4*** | ***27*** |  |
