# Supplementary material for: Contextual adaptation of the Personnel Evaluation Standards for assessing faculty evaluation systems in developing countries: the case of Iran
Source: BMC Med Educ. 2009 Apr 28;9:18. doi: 10.1186/1472-6920-9-18 (PMC2680845; doi:10.1186/1472-6920-9-18)
Supplement: Additional file 2 — A schematic view of the survey instrument. [file 1472-6920-9-18-S2.doc]

### Additional file 2: A schematic view of the Instrument

**Part I:**

| **Demographic and Academic information:** | | |
| --- | --- | --- |
| Department of affiliation… | Age...…year  Gender: Female Male | |
| Academic Rank:   Professor  Associate Professor Assistant Professor  Lecturer | The subject of your administrative appointment: … | Experience…*year* |
| Employment Position:  Permanent probationary  None-permanent Other… | Subject of your academic administrative position: … | |

**Part II:**

Example of the scales with the statement of the Standard and five domains of faculty members’ roles

*Please choose one answer that best matches your view on the statement given*

| **Never** | **Occasionally** | **Frequently** | **Always** | **Don’t know** |
| --- | --- | --- | --- | --- |
| 1 | 2 | 3 | 4 | 0 |

| **No.** | **Statement of the adapted standard** | **Faculty Member Role** | | | | |
| --- | --- | --- | --- | --- | --- | --- |
|  |  | **Teaching** | **Research** | **Clinical & healthcare Service** | **Administration** | **Self-development** |
| 1 |  |  |  |  |  |  |
|  |  |  |  |  |  |  |
|  |  |  |  |  |  |  |
|  |  |  |  |  |  |  |
| 27 |  |  |  |  |  |  |

**Part III:**

Open-ended questions
